# A Policy and Practice Review of Consumer Protections and Their Application to Hospital-Sourced Data Aggregation and Analytics by Third-Party Companies

**DOI:** 10.3389/fdata.2020.603044

**Published:** 2021-02-12

**Authors:** Vasiliki Rahimzadeh

**Affiliations:** Stanford Center for Biomedical Ethics, Stanford University, Stanford, CA, United States

**Keywords:** data aggregation, EHR, privacy, HIPAA, California Consumer Privacy Act, Proposition 24

## Abstract

The Office of the National Coordinator for Health Information Technology estimates that 96% of all U.S. hospitals use a basic electronic health record, but only 62% are able to exchange health information with outside providers. Barriers to information exchange across EHR systems challenge data aggregation and analysis that hospitals need to evaluate healthcare quality and safety. A growing number of hospital systems are partnering with third-party companies to provide these services. In exchange, companies reserve the rights to sell the aggregated data and analyses produced therefrom, often without the knowledge of patients from whom the data were sourced. Such partnerships fall in a regulatory grey area and raise new ethical questions about whether health, consumer, or health and consumer privacy protections apply. The current opinion probes this question in the context of consumer privacy reform in California. It analyzes protections for health information recently expanded under the California Consumer Privacy Act (“CA Privacy Act”) in 2020 and compares them to protections outlined in the Health Information Portability and Accountability Act (“Federal Privacy Rule”). Four perspectives are considered in this ethical analysis: 1) standards of data deidentification; 2) rights of patients and consumers in relation to their health information; 3) entities covered by the CA Privacy Act; 4) scope and complementarity of federal and state regulations. The opinion concludes that the CCPA is limited in its application when health information is processed by a third-party data aggregation company that is contractually designated as a business associate; when health information is deidentified; and when hospital data are sourced from publicly owned and operated hospitals. Lastly, the opinion offers practical recommendations for facilitating parity between state and federal health data privacy laws and for how a more equitable distribution of informational risks and benefits from the sale of aggregated hospital data could be fostered and presents ways both for-profit and nonprofit hospitals can sustain patient trust when negotiating partnerships with third-party data aggregation companies.

## Introduction

Less is certainly not more when aggregation of quality hospital system data is concerned. Indeed, aggregation puts the “big” in big data. Aggregation refers to the semantic integration of datasets from disparate sources, sizes, and elements into a shareable format. It allows for cross-system analyses of hospital trends shown to reduce medical error, inform safer therapies, and enable timely public health reporting (Fefferman et al., 2005), to name but a few applications (Olsen et al., 2007). New machine learning and artificial intelligence applications in healthcare likewise depend on robust data aggregation for training algorithms to automate certain care delivery tasks with precision and effectiveness (Char et al., 2018). While these data are primarily aggregated through extraction from electronic health records (EHR), and follow a complex trajectory from the point of care to aggregation (Rolnick, 2013), problems with EHR network interoperability largely persist across U.S. hospitals despite regulatory reforms to improve their meaningful use in 2009 (United States Congress, 2009) and again in 2016 (21st Century Cures Act, 2016).

Hospitals are handicapped in performing aggregation in-house due, in large part, to limited availability of EHR-based rather than insurance claims-based data, exceedingly high administrative costs of producing datasets, and technological limitations involving software (4). A growing market for third-party data aggregation services is poised to fill critical infrastructural gaps that federal agencies have been thus far slow to fill (Wang et al., 2017; Groves et al., 2013; Challenge.gov, 2009). Optum One, for instance, describes their data aggregation services as “source- and vendor-agnostic,” meaning the company integrates claims, clinical, sociodemographic, genetic, and care management data—herein referred to as hospital data—to identify population-level patterns irrespective of the record platform from which the data originated.

Analyses performed on the aggregate data can be subsequently fed back to the hospitals to inform quality improvement, clinical teaching, and research, among others (Fefferman et al., 2005). Third-party data aggregation companies (TDAC) reserve the right to sell the aggregate data for marketing and other commercial purposes, provided that the data are appropriately protected. Health data (e.g., from EHRs, insurance claims databases, and genetic data) are distinct from other common consumer data types (e.g., credit card numbers, geolocation, and demographic data). The use and disclosure of protected health information are governed federally by the Health Information Portability and Accountability Act (HIPAA, herein referred to as the Federal Privacy Rule), while the Federal Trade Commission has jurisdiction over consumer data. Since 2018, three states have also passed their own Internet consumer privacy legislation in California, Nevada, and Maine (National Conference of State Legislatures, 2009).

Though the legislations differ in scale and scope, they broadly aim to strengthen the rights of consumers to decide what, how, and with whom their personal information is shared. These rights and protections applied exclusively to consumer data until Californians voted to approve Proposition 24 during the latest State Elections in November 2020. Among other amendments, Proposition 24 expanded protections outlined in the existing California Consumer Privacy Act (herein referred to as the “CA Privacy Act”) to include health information as a special category of sensitive personal information and “the unauthorized use or disclosure of which creates a heightened risk of harm to the consumer” (State of California, 2018).

The expanded protections blur the neat legislative distinction between personal and health information protections under the CA Privacy Act. As Price aptly notes, health information held by entities outside the Federal Privacy Rule’s ambit “might seem to improve the problem of data fragmentation; these entities can gather data unhindered by HIPAA’s strictures. On the other hand, fragmentation may increase because different entities, with different forms of health data, are governed by different legal regimes” (Price, 2018).

Greater involvement of third-party aggregation of hospital-sourced data prompts asking whether individuals are patients, consumers, or both under applicable privacy laws and raises new ethical questions about what rights individuals have in the emerging medical datasphere (Béranger, 2016). It is unclear, for example, if contractual relationships between data aggregation companies and hospitals or the aggregation tasks a company performs determine which privacy regimes should apply. Reflections on these questions regarding patient rights to know whether and how their data are shared could have broader implications if other states follow suit in expanding special consumer protections to health information.

This opinion probes these questions through a close reading of the expanded protections for health information in the CA Privacy Act. Specifically, it analyzes the protections afforded to hospital-sourced data aggregated by TDACs from four ethical perspectives: 1) standards of data deidentification to minimize informational risk; 2) rights of patients and consumers in relation to their health information; 3) entities covered by the CA Privacy Act; 4) and scopes of Federal and State regulations. The opinion concludes with practical recommendations for how to achieve a more equitable distribution of informational risks and benefits from the sale of aggregated hospital data and ways to sustain patient trust in private-private partnerships between hospitals and TDACs.

### Deidentification Requirements: Separate but Equal?

Both the Federal Privacy Rule and the CA Privacy Act acknowledge that certain types of data merit special protection and generally agree on the inherent characteristics that make health information identifying. The Federal Privacy Rule explicitly governs the use and disclosure of identifying health information, termed protected health information, while the CA Privacy Act protects much broader categories of identifiable personal information. The main goal of the Federal Privacy Rule is to “assure that individuals’ health information is properly protected while allowing the flow of health information needed to provide and promote high quality health care and to protect the public’s health and well being … Given that the health care marketplace is diverse, the Rule is designed to be flexible and comprehensive to cover the variety of uses and disclosures that need to be addressed” (Department of Health and Human Services, 2003).

The Federal Privacy Rule and the CA Privacy Act both exempt deidentified data. Additionally, the CA Privacy Act exempts protected health information that is used and disclosed by covered entities and business associates subject to the Federal Privacy Rule. Together, these exemptions allow for deidentified health data to be securely and efficiently exchanged for quality improvement purposes, approved health research and public health management, and many other uses. It is important to note that aggregate datasets can include readily identifiable, coded (i.e., personal identifiers are linked to the data by secure keys held by those processing the data), and deidentified (i.e., irreversibly delinked) information.

The Federal Privacy Rule applies prescriptive standards for determining when protected health information is appropriately deidentified, whereas the CA Privacy Act applies a reasonableness standard. The Federal Privacy Rule requires that information must be stripped of 18 unique identifiers to be deemed deidentified, termed the safe harbor rules, or otherwise verified by a field expert. The prescriptiveness of the Federal Privacy Rule leaves little room for interpretation and therefore can be more consistently applied across health systems, providers, and research institutions.

The CA Privacy Act, in contrast, applies the Federal Trade Commission’s proposed reasonability standard for deidentification. This standard requires that to be deidentified, data “cannot reasonably identify, relate to, describe, be capable of being associated with, or be linked, directly or indirectly, to a particular consumer.” Reasonableness is both data- and context-specific and, as a result, interpretable. That is, some types of data carry a higher likelihood of harm resulting from reidentification depending on how they are shared and with whom. The requirement for deidentification under the CA Privacy Act thus transcends specific categories and does not adopt predetermined methods to fulfill the deidentification requirement. The reasonability standard allows for deidentification to be determined in relation to actual environments in which data are exchanged and their associated risks. In this way, the reasonability standard can tailor deidentification methods to the specific data use and can be more flexible to emerging advances in privacy-preserving technologies and accountability policies, where applicable.

Could TDACs achieve comparable protections for sensitive personal information, e.g., health information under the CA Privacy Act? Several scenarios are possible. TDACs, other businesses, and data brokers subject to the CA Privacy Act could adopt the HIPAA safe harbor rules or apply the expert determination method to deidentify health information. In this case, health information used and shared by TDACs would be protected using the same deidentification standards as if it were managed by a HIPAA-covered entity. Alternatively, companies could apply stricter deidentification requirements and therefore grant patients additional protection compared to what is federally required. This could be the case if a TDAC demonstrates the health information it aggregates can still be reasonably reidentified despite applying the safe harbor or expert determination methods. Finally, there is the possibility that companies could exploit the flexibility built into the reasonability standard and adopt weaker deidentification practices, making health information less secure under the CA Privacy Act.

Data protection scholars and ethicists alike agree that deidentification is a spectrum and not a uniform standard (Stalla-Bourdillon and Wu, 2019). Indeed, some types of inherently identifying health information (e.g., genetic data (Homer et al., 2008)) pose challenges to the efficacy of both the reasonability standard and prescriptive approaches to deidentification. As reidentification becomes more “reasonable” with advanced information technologies (Kulynych and Greely, 2017), prescriptive deidentification strategies can quickly become outdated. So while the Privacy Rule applies deidentification standards consistently, those standards can underprotect particularly sensitive types of health information. The reasonability standard may better tailor data protections to the unique sensitivities and risks of disclosure, but its flexibility can mean that protections are applied inconsistently across the various entities which collect, use, and share this information. The next section explains the case when TDACs are contractually obligated to adopt the Federal Privacy Rule’s more granular deidentification standard for hospital data.

### The Business Association Designation: Health Insurance Portability and Accountability Act

One regulatory pathway by which TDACs can use and disclose protected health information is to serve as a “business associate” of a HIPAA-covered entity. Covered entities can include healthcare providers, health plans, or healthcare clearinghouses. TDACs could receive protected health information from hospitals prior to aggregation and subsequently deidentify it on behalf of the HIPAA-covered entity as part of a business associate agreement provided that they apply the expert determination standard or the safe harbor rules. The Department of Health and Human Services also recognizes “data aggregation” among the qualified services a TDAC could perform under a special type of business associate’s agreement (Department of Health and Human Services, 2008), called a health information organization (HIO). The HIO designation permits also TDACs to, among other things, provide data aggregation services related to the healthcare operations of the covered entities for which it has agreements.

Both patients and companies have the potential to benefit from data aggregation partnerships. Hospitals can better serve patients through monitoring quality, safety, and provider performance data that TDACs make available. TDACs benefit financially from providing aggregation services and selling trend analyses not only to individual hospitals they may partner with directly but also to researchers and other companies. These revenues allow companies to invest in new information technologies that further expand the services they can provide to hospital systems within their network. The Federal Privacy Rule permits also TDACs to share deidentified data beyond the healthcare operations.

There is growing ethical concern about the emergence of new markets for aggregated hospital data and how companies may take advantage of regulatory loopholes to bypass consent from patients themselves. A TDAC that contracts with a hospital as a business associate can legally receive health information from the covered entity, deidentify it, and sell the deidentified data in the aggregate as well as any resulting trend analyses for the company’s own commercial gain without patient authorization. Individuals treated at hospitals which partner with TDACs are often unaware that such partnerships exist and that their protected health information—albeit deidentified—is being sold by third-party companies for commercial purposes in many cases (Price et al., 2019).

Deidentified hospital data can be sold without a patient’s authorization under a TDAC’s business associate agreement; however, patients may have the option to invoke their right to an accounting of disclosures to better understand with whom their protected health information has been shared. The Federal Privacy Rule permits individuals under 45 CFR § 164.528 to obtain a record of certain disclosures of their protected health information by covered entities or their business associates, including TDACs where applicable. Covered entities and business associates are required to account for any and all disclosures of an individual’s protected health information unless it was to carry out treatment, payment, and healthcare operations; it was for national security or intelligence purposes or related to correctional institutions or law enforcement officials; it was part of a limited dataset or occurred prior to the compliance date (April 2003). Requesting an accounting of disclosures could allow patients some transparency about existing partnerships between the hospital and any third-party companies it contracts with to manage protected health information if unknown to patients at the time of care (See Supplementary Material).

Hospitals are also not obligated to use the data TDACs aggregate for quality improvement. Hospitals also cannot condition the future sale of this data on such improvement. Importantly, neither the covered entity that contracts with the HIO or the HIO itself is liable if a violation of the Federal Privacy Rule is discovered and an appropriate business associate agreement is in place. The HIO is instead required to report any noncompliance with the agreement terms to the covered entity. A covered entity is moreover not required to oversee HIO compliance but must act to address the noncompliance when disclosed or else terminate the agreement. Accountability for patient privacy, therefore, rests on 1) elective disclosure of noncompliance by the HIO and 2) swift action on the part of hospitals to cure the noncompliance, and liability for the privacy violation remains ambiguous. While permissible under a recognized business associate agreement, there is a chance the sale and exchange of aggregate hospital data could disproportionately benefit companies. Patients, in turn, assume the informational risks associated with having their data aggregated and sold with limited ability to share directly in the benefits. Consumer data protections, in contrast, may afford greater agency in the sale of personal information that in the future could include more categories of health data. The following section illustrates how through discussing recent reforms to consumer data protections in California.

### Data Brokering under the CA

**Privacy Act**

The CA Privacy Act was introduced in 2018 as a state-wide legislation to afford California consumers more control over personal information that businesses and data brokers collect about them (See Supplementary Material). Assembly Bill No. 375 effectively enacted the CA Privacy Act on January 1, 2020, and grants consumers four primary rights:

1 Right to know: A consumer may request that a business disclose 1) categories of personal information it collects about them, 2) the sources of that information, 3) the business purposes for collecting or selling the information, and 4) third parties with which the information is being shared.
2 Right to delete: A consumer may request that a business deletes personal information and requires businesses to follow through on a verified request.
3 Right to opt out: A consumer’s may direct a business not to sell their personal information at any time.^1^
4 Right to nondiscrimination: A business shall not discriminate against a consumer because they exercised their rights under the Act.

The CA Privacy Act further obligates the business with revenues greater than $15M to process data from more than 50,000 individuals, ^2^ households, or devices, where more than 50% of revenues are derived from the sale of information to assure consumers it has no intent to reidentify the data; has implemented technical safeguards and processes to prohibit reidentification; has taken necessary steps to prevent inadvertent release of deidentified data.

While it covers all uses, disclosures, and management of protected health information, the Federal Privacy Rule is not presumed to complement state-based consumer data protections. Many hospitals are designated not-for-profit institutions or designated as a HIPAA-covered entity and therefore exempt from the CA Privacy Act. According to the California Department of Health and Human Services, 56 (7%) of the 492 registered acute care hospitals in California are governed by for-profit corporations (California Department of Health and Human Services Facilities List Data, 2021).

1 the entity meets the criteria designating them a “business” or “data broker”;
2 they are not a regulated entity that manages patient information according to HIPAA or California Medical Information Act regulations;
3 the data collected, used, shared, or sold are “reasonably identifiable” (Noordyke, 2020);
4 data include financial account information, racial or ethnic origin, religious beliefs, union membership, sexual orientation, genetic data, and precise geolocation data.

The consumers, i.e., patients about whom TDACs broker personal information, may be able to exercise additional consumer data privacy rights in some states where for-profit hospitals operate based in part on their federal compliance, nature, and type of data brokering activities and identifiability of the aggregate data.

**FIGURE 1:**
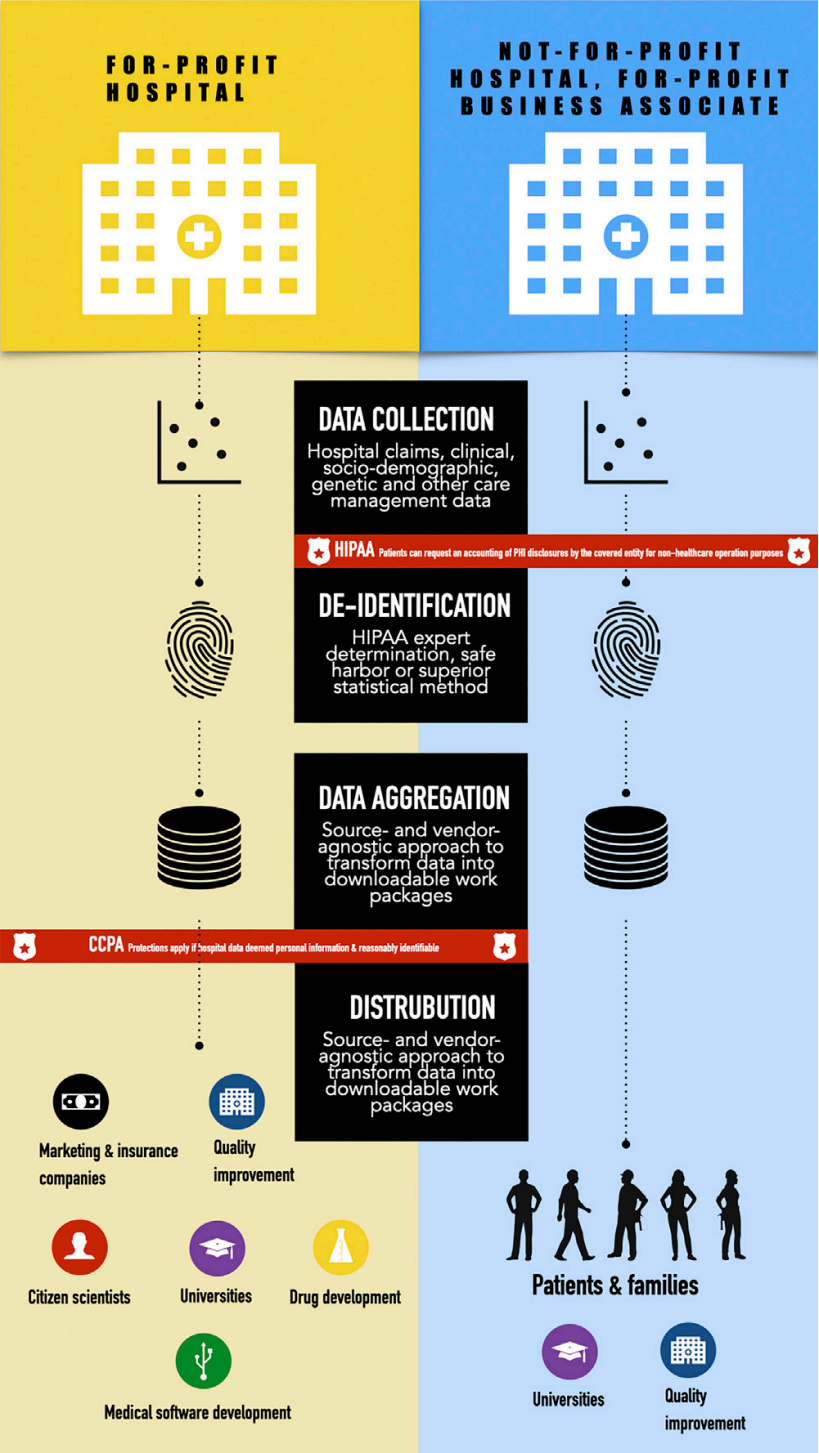
Comparison of applicable health and consumer data protections between for-profit hospitals and non-for-profit hospitals with for-profit business associates.

A close reading of the CA Privacy Act in conversation with the Federal Privacy Rule reveals that TDACs which operate without a business associate agreement and aggregate “reasonably” identifiable health information are liable under the CA Privacy Act. Patients can also exercise the four rights to know, correct, delete, and opt out of the sale of their personal information described above (Figure 1). Operationalizing these rights is not without specific logistical and feasibility challenges in the ways patients are informed about how their data are used/shared with TDACs. The delivery and timing of this information could be especially fraught in an emergency or other serious clinical situations in which patients may not be fully able to appreciate the short- and long-term implications of what types of data will be aggregated and sold nor able to navigate the digital minefield that is submitting a verified opt-out request.

## Recommendations

Complementary protections at the federal and state levels is essential for sustaining public trust with patients and consumers, particularly if more states follow California’s lead. More explicit federal and state guidance is therefore needed regarding the nature and scope of data aggregation activities TDACs can perform using hospital-sourced information. First, the Office of the National Coordinator could consider narrowing permissions for how TDACs access, use, and disclose aggregate hospital data for which existing deidentification methods may be insufficient, for example, involving data that are particularly identifying or stigmatizing. Second, the National Coordinator should work more closely with state legislatures in the process of drafting consumer privacy legislation that propose to include health information to ensure complementarity. TDACs should consider, for example, applying the safe harbor, expert determination, or a superior method of deidentification to achieve complementarity with the Federal Privacy Rule.

Finally, more changes to the interplay of state and federal privacy protections for health information are expected following the approval of Proposition 24, otherwise called the California Privacy Rights and Enforcement Act. The revised CA Privacy Act in California is set to come into full force on January 1, 2023. It grants the state and California businesses new powers that have important implications for implementing expanded protections for “sensitive” personal information, specifically health and genetic information (Table 1). Proposition 24 carves out funding for a new agency that will oversee the amended CA Privacy Act enforcement to issue penalties and manage all consumer correction/deletion/opt-out requests. Businesses are also permitted to pass on a portion of the cost for complying with the expanded CA Privacy Act onto consumers. Indeed, the American Civil Liberties Union opposed Proposition 24 primarily for this reason. The new enforcement agency should therefore consider placing caps on how much companies can charge for stricter privacy protections, if not eliminate them outright. Capping the amount companies can pass on to consumers helps avoid establishing a pay-for-privacy precedent that discriminates against lower socioeconomic groups.

**TABLE 1:** Section 10 regarding use and sale of “sensitive” information added to the California Consumer Privacy Act following vote to approve Proposition 24 in November 2020.

SEC. 10. Section 1798.121 is added to the Civil Code, to read: 1798.121. Consumers’ Right to Limit Use and Disclosure of Sensitive Personal Information 1798.121
(a) **A consumer shall have the right, at any time, to direct a business that collects sensitive personal information about the consumer to limit its use of the consumer’s sensitive personal information to that use which is necessary to perform the services or provide the goods** reasonably expected by an average consumer who requests those goods or services, to perform the services set forth in paragraphs (2), (4), (5), and (8) of subdivision (e) of Section 1798.140, and as authorized by regulations adopted pursuant to subparagraph (C) of paragraph (19) of subdivision (a) of Section 1798.185. A business that uses or discloses a consumer’s sensitive personal information for purposes other than those specified in this subdivision shall provide notice to consumers, pursuant to subdivision (a) of Section 1798.135, that this information may be used or disclosed to a service provider or contractor, for additional, specified purposes and that consumers have the right to limit the use or disclosure of their sensitive personal information
(b) **A business that has received direction from a consumer not to use or disclose the consumer’s sensitive personal information**, except as authorized by subdivision (a), shall be prohibited, pursuant to paragraph (19) of subdivision (c) of Section 1798.135, from using or disclosing the consumer’s sensitive personal information for any other purpose after its receipt of the consumer’s direction unless the consumer subsequently provides consent for the use or disclosure of the consumer’s sensitive personal information for additional purposes
(c) **A service provider or contractor that assists a business in performing the purposes authorized by subdivision (a) may not use the sensitive personal information after it has received instructions from the business and to the extent, it has actual knowledge that the personal information is sensitive personal information for any other purpose.** A service provider or contractor is only required to limit its use of sensitive personal information received pursuant to a written contract with the business in response to instructions from the business and only with respect to its relationship with that business
(d) **Sensitive personal information that is collected or processed without the purpose of inferring characteristics about a consumer is not subject to this section**, as further defined in regulations adopted pursuant to subparagraph (C) of paragraph (19) of subdivision (a) of Section 1798.185, and shall be treated as personal information for purposes of all other sections of this act, including Section 1798.100

## Conclusion

Data aggregation is a necessary yet time- and technology-intensive task in making health systems safer, more effective, and less expensive by analyzing hospital data in EHRs. Third-party data aggregation companies are increasingly filling unmet needs in this regard but complicate the data protection landscape where health and consumer data protection could simultaneously apply in a growing market for hospital data. The current opinion presents an ethical comparison of these protections outlined in the CA Privacy Act and Federal Privacy Rule from four primary perspectives: 1) standards of data deidentification; 2) rights of patients and consumers in relation to their health data; 3) entities covered by the acts; 4) scopes of regulation.

The first version of the CA Privacy Act introduced landmark consumer privacy legislation in 2018. It applied to certain businesses and data brokers that met certain revenue (more than $15M) and data processing (more than 50,000 consumers, households, or devices) criteria. Yet, businesses and data brokers were able to circumvent some restrictions on the “sale” of information, for example, and imposed the same requirements on all categories of personal information irrespective of differences in sensitivity. Patients and consumers about whom health information, in particular, was systematically collected and sold were disadvantaged given the heightened sensitivity of this information and ease with which it could be readily linked with other public sources.

Consumer privacy rights can be triggered when a TDAC is not contractually designated as a business associate with covered entity aggregates health information that can be “reasonably” identifiable. Moreover, the CA Privacy Act protections could apply to hospital data sourced from privately owned and operated hospitals and sold to other businesses, entities, or data brokers subject to the CA Privacy Act. The expanded protection for health information fills a regulatory gap left open by the Federal Privacy Rule and, as a result, strengthens protection for patients treated at for-profit hospitals and consumers of health-related services such as direct-to-consumer genetic testing.

When TDACs operate as a business associate of a covered entity, patients could exercise their request for an accounting of disclosures for nonhealthcare operation purposes to better understand with whom their protected health information has been shared. Enhanced representation from patient groups in business associate negotiations is one approach to establishing a more equitable benefit-sharing structure that prioritizes patient care and financing of patient-led programs from revenues received from a partnership with TDACs. Further empirical research is needed to understand what, if any, patient privacy and other ethical interests should be factored into decisions to partner with third-party aggregation companies from the perspectives of patients and hospital administrators.

## Footnotes

1 In addition, the CA Privacy Act includes a special opt-in clause for the sale and brokering of personal health information from minors. A business is not permitted to sell personal information from consumers younger than 16 years of age unless a consumer older than 13 but less than 16 or their guardian explicitly consents to the sale.

2 Passage of Proposition 24 following the 2020 Elections increased this threshold to 100,000.

## Abbreviations

CCPA, California Consumer Privacy Act (referred to as the “CA Privacy Act”); CPRA, California Privacy and Enforcement Act (i.e., Proposition 24); EHR, electronic health record; HIO, health information organization; HIPAA, Health Information Portability and Accountability Act (referred to as the “Federal Privacy Rule”); TDAC, third-party data aggregation company.
