# Supplementary material for: A Policy and Practice Review of Consumer Protections and Their Application to Hospital-Sourced Data Aggregation and Analytics by Third-Party Companies
Source: Front Big Data. 2021 Feb 12;3:603044. doi: 10.3389/fdata.2020.603044 (PMC7931961; doi:10.3389/fdata.2020.603044)
Supplement: Supplementary file 1 [file table1.docx]

**Box 1**— Glossary of key terms from the Health Insurance Portability and Accountability Act. Relevant clauses with respect to data aggregation services are bolded (emphasis added).

| **Accounting of disclosures** | **Information** that describes a covered entity's disclosures of PHI other than for treatment, payment, and health care operations; disclosures made with Authorization; and certain other limited disclosures. For those categories of disclosures that need to be in the accounting, the accounting must include disclosures that have occurred during the 6 years (or a shorter time period at the request of the individual) prior to the date of the request for an accounting. |
| --- | --- |
| **Business associate** | A person or entity, other than a member of the covered entity’s work force, that   1. Creates, receives, maintains, or transmits **protected health information** (PHI) for a function or activity (e.g. claims processing or administration, **data analysis**, processing or administration, utilization review, quality assurance, patient safety) for or on behalf of a covered entity or 2. Provides services (e.g. legal, actuarial, accounting, consulting, **data aggregation,** management, administrative, accreditation, or financial) for such covered entity, or to or for an organized health care arrangement in which the covered entity participates, where the **provision of the service involves the disclosure of protected health information** |
| **Covered entity** | 1. A health plan 2. A health care clearinghouse 3. A health care provider who transmits any health information in electronic form |
| **Data aggregation** | The **combining** of protected health information by the business associate with the protected health information received by the business associate in its capacity as a business associate of another covered entity, to permit **data analyses** that relate to the **health care** **operations** of the respective covered entities |
| **Expert determination** | Certification by a person with appropriate knowledge of and experience with generally accepted statistical and scientific principles and m**ethods for rendering information not individually identifiable** that there is a very small risk that the information could be used by the recipient to identify the individual who is the subject of the information, alone or in combination with other reasonably available information. The person certifying statistical de-identification must document the methods used as well as the result of the analysis that justifies the determination. A covered entity is required to keep such certification, in written or electronic format, for at least 6 years from the date of its creation or the date when it was last in effect, whichever is later. |
| **Healthcare operations** | Any of the following activities of the covered entity to the extent that the activities are related to covered functions:   - 1. Conducting **quality assessment and improvement activities**, including outcomes evaluation and development of clinical guidelines, provided that the obtaining of generalizable knowledge is not the primary purpose of any studies resulting from such activities; patient safety activities; population-based activities relating to improving health or reducing health care costs, protocol development, case management and care coordination, contacting of health care providers and patients with information about treatment alternatives; and related functions that do not include treatment;   2. Reviewing the competence or qualifications of health care professionals, evaluating practitioner and provider performance, health plan performance, conducting training programs in which students, trainees, or practitioners in areas of health care learn under supervision to practice or improve their skills as health care providers, training of non-health care professionals, accreditation, certification, licensing, or credentialing activities;   3. Underwriting, enrollment, premium rating, and other activities related to the creation, renewal, or replacement of a contract of health insurance or health benefits, and ceding, securing, or placing a contract for reinsurance of risk relating to claims for health care (including stop-loss insurance and excess of loss insurance)   4. Conducting or arranging for medical review, legal services, and auditing functions, including fraud and abuse detection and compliance programs;   5. Business planning and development, such as conducting cost-management and planning-related analyses related to managing and operating the entity, including formulary development and administration, development or improvement of methods of payment or coverage policies; and   6. Business management and general administrative activities of the entity, including, but not limited to: - Management activities relating to implementation of and compliance with the requirements of this subchapter; - Customer service, including the provision of **data analyses** for policy holders, plan sponsors, or other customers, provided that **protected health information is not disclosed** to such policy holder, plan sponsor, or customer. - Resolution of internal grievances; - The sale, transfer, merger, or consolidation of all or part of the covered entity with another covered entity, or an entity that following such activity will become a covered entity and due diligence related to such activity; and   1. Creating **de-identified health information** or a **limited data set**, and fundraising for the benefit of the covered entity. |
| **Protected health information** | Any individually identifiable **health information** held or transmitted by a covered entity or its business associate, in any form or media, whether electronic, paper, or oral. |
| **Safe Harbor** | \| The following identifiers must be removed to be compliant with this de-identification method for covered entities and their business associates:   1. Names 2. All geographic subdivisions smaller than a state, including street address, city, county, precinct, ZIP Code, and their equivalent geographical codes, except for the initial three digits of a ZIP Code if, according to the current publicly available data from the Bureau of the Census:    1. The geographic unit formed by combining all ZIP Codes with the same three initial digits contains more than 20,000 people.    2. The initial three digits of a ZIP Code for all such geographic units containing 20,000 or fewer people are changed to 000. 3. All elements of dates (except year) for dates directly related to an individual, including birth date, admission date, discharge date, date of death; and all ages over 89 and all elements of dates (including year) indicative of such age, except that such ages and elements may be aggregated into a single category of age 90 or older. \|  \| 1. Telephone numbers. 2. Facsimile numbers. 3. Electronic mail addresses. 4. Social security numbers. 5. Medical record numbers. 6. Health plan beneficiary numbers. 7. Account numbers. 8. Certificate/license numbers. 9. Vehicle identifiers and serial numbers, including license plate numbers. 10. Device identifiers and serial numbers. 11. Web universal resource locators (URLs). 12. Internet protocol (IP) address numbers. 13. Biometric identifiers, including fingerprints and voiceprints. 14. Full-face photographic images and any comparable images. 15. Any other unique identifying number, characteristic, or code, unless otherwise permitted by the Privacy Rule for re-identification. \| \| --- \| --- \| --- \| |

**Box 2**— Glossary of key terms in the California Consumer Privacy Act.

| **Aggregate consumer information** | Information that relates to a group or category of consumers, from which individual consumer identities have been removed, that is not linked or reasonably linkable to any consumer or household, including via a device. “Aggregate consumer information” does not mean one or more individual consumer records that have been de­identified. |
| --- | --- |
| **Business** | For-profit entity that  i) Does business in California  ii) Collects the personal information (“PI”) of California residents, and  iii) Maintains annually gross revenues of $25 million or more; (b) derive half or more of their annual revenue from selling PI; or (c) transact in the PI of 50,000 or more consumers, households, or devices per year. |
| **Consumer** | A natural person who is a California resident, as defined in Section 17014 of Title 18 of the California Code of Regulations, as that section read on September 1, 2017, however identified, including by any unique identifier. |
| **Data broker** | A business that knowingly collects and sells to third parties the personal information of a consumer with whom the business does not have a direct relationship. |
| **De-identified** | Information that cannot reasonably identify, relate to, describe, be capable of being associated with, or be linked, directly or indirectly, to a particular consumer, provided that a business that uses deidentified information:  (1) Has implemented technical safeguards that prohibit reidentification of the consumer to whom the information may pertain.  (2) Has implemented business processes that specifically prohibit reidentification of the information.  (3) Has implemented business processes to prevent inadvertent release of deidentified information.  (4) Makes no attempt to reidentify the information. |
| **Sale** | Selling, renting, releasing, disclosing, disseminating, making available, transferring, or otherwise communicating orally, in writing, or by electronic or other means, a consumer’s personal information by the business to another business or a third party for monetary or other valuable consideration |
| **Service Provider** | A sole proprietorship, partnership, limited liability company, corporation, association, or other legal entity that is organized or operated for the profit or financial benefit of its shareholders or other owners, that processes information on behalf of a business and to which the business discloses a consumer’s personal information for a business purpose pursuant to a written contract, provided that the contract prohibits the entity receiving the information from retaining, using, or disclosing the personal information for any purpose other than for the specific purpose of performing the services specified in the contract for the business, or as otherwise permitted by this title, including retaining, using, or disclosing the personal information for a commercial purpose other than providing the services specified in the contract with the business. |
| **Personal Information** | Personal information is information that identifies, relates to, or could reasonably be linked with you or your household. |
